# Clinical and Demographical Characteristics of Patients with Medication Overuse Headache in Argentina and Chile: Analysis of the Latin American Section of COMOESTAS Project

**DOI:** 10.1186/s10194-015-0561-1

**Published:** 2015-09-18

**Authors:** Beatriz Shand, Maria Teresa Goicochea, Raul Valenzuela, Ricardo Fadic, Rigmor Jensen, Cristina Tassorelli, Giuseppe Nappi, the COMOESTAS CONSORTIUM

**Affiliations:** Department of Neurology, Pontificia Universidad Católica of Chile, Santiago, Chile; Integral Pain Centre, Fundación para la Lucha contra las Enfermedades Neurológicas Infantiles (FLENI), Buenos Aires, Argentina; Danish Headache Centre, Glostrup Amtssygehuset, University of Copenhagen, Copenhagen, Denmark; Headache Science Centre, C. Mondino National Neurological Institute, Pavia, Italy; Department of Brain and Behavioral Sciences, University of Pavia, Pavia, Italy

**Keywords:** Medication Overuse Headache (MOH), Chronic headache, Ergotamine

## Abstract

**Background:**

Data on the characteristics of Medication Overuse Headache (MOH) in Latin American (LA) are scarce. Here we report the demographic and clinical features of the MOH patients from Argentina and Chile enrolled in the multinational COMOESTAS project in the period 2008–2010.

**Methods:**

The LA population was formed by 240 MOH subjects, 110 from Chile and 130 from Argentina, consecutively attending the local headache centres. In each centre, specifically trained neurologist interviewed and confirmed the diagnosis according to the ICHD-II criteria. A detailed history was collected on an electronic patient record form.

**Results:**

The mean patient age was 38.6 years, with a female/male ratio of 8:2. The mean time since onset of the primary headache was 21 years, whereas duration of MOH was 3.9 years. The primary headache was migraine without aura in 77.5 % and migraine with aura in 18.8 %. Forty two % of the patients self-reported emotional stress associated with the chronification of headache; 43.8 % reported insomnia. The most overused medications were acute drug combinations containing ergotamine (70 %), NSAIDs (33.8 %) and triptans (5.4 %).

**Conclusion:**

Though little described, MOH is present also in LA, where it affects mostly women, in the most active decades of life. Some differences emerge as regards the demographic and clinical characteristics of MOH in this population as compared to Europe or Northern America. What seems more worrying about MOH in Argentina and Chile is that most patients overuse ergotamine, a drug that may cause serious adverse events when used chronically. These findings once more underscore the importance of properly diagnose and treat MOH.

## Background

Medication overuse headache (MOH) is defined by the chronification of an episodic headache in association with the excessive use of any type of symptomatic medication. It has been recognized for over 50 years [1]. It primarily affects women (3.5:1), with an average age of 40 and is usually diagnosed several years after overuse has started [2, 3]. In the vast majority of patients, it is possible to recognize a previous primary headache, in most cases represented by migraine [4, 5]. Triptans and non steroid anti-inflammatory drugs (NSAIDs) are the main prescription drugs overused in Western Europe [6, 7]. Some risk factors have been proposed for MOH: high frequency of both pain medication use and pain episodes, use of two or more different medications, coexistence of other body pains, high levels of daily stress, low socio-economic status, psychiatric comorbidities [1, 8, 9]. European epidemiological studies have shown a prevalence of 1 to 3 % [10–12], but in specialized headache centres the percentage markedly increases [13, 14]. Precise epidemiological data about the prevalence of MOH in Latin Americaare lacking. In Chile, for instance, though the condition has been known for many years [15], there is only one publication investigating the epidemiology of chronic headache, but not focused on MOH [16]. In this paper we present the main demographic and clinical characteristics of MOH in a population of 240 MOH sufferers living in Argentina and Chile.

## Methods

We evaluated 240 patients consecutively visited in the Neurology Department of Pontificia Universidad Católica de Chile (PUC) and in the Integral Pain Center at the Fundación para la Lucha contra las Enfermedades Neurológicas Infantiles (FLENI), Argentina, from January 2008 to December 2010 by neurologists who underwent the specific training for MOH diagnosis developed within the COMOESTAS project [17, 18]. Both LA institutions take care of privately insured patients. Most of the patients included in the protocol were recruited during spontaneous headache consultation at the outpatient clinics both centers (98.3 %). Very few patients were recruited while hospitalized. As patients were enrolled before the publication of ICDH-IIIbeta [19], the diagnosis of primary headaches (migraine, chronic migraine and tension-type headache) and MOH was made on ICHDII [20] and ICHDII-R criteria [21]. For the scopes of the present paper, diagnoses were then reviewed and confirmed retrospectively using the ICHDIII beta criteria.

Demographic data, habits, personal and family medical history were recorded, as well as MOH history, MOH characteristics and medications being overused. All the information included in this paper was obtained through a thorough anamnestic report.

The data were collected within the activities of the COMOESTAS project, which obtained the approval of the Research Ethics Committee at the Medical School of Pontificia Universidad Católica de Chile and the Ethics Committee and Biomedical Research at FLENI. Written consent was obtained for all patients.

### Statistical analysis

The categorical variables were described as absolute and relative frequency. The continuous variables were described as mean and standard deviation. The level of significance was set to p < 0.05.

All of the analyses were done with the Program: SPSS Inc. Released 2008. SPSS Statistics for Windows, Version 17.0. Chicago: SPSS Inc.

## Results

Of the 240 patients evaluated, 110 were enrolled in Chile and 130 in Argentina. The mean age was 38.7 years (range: 18–72), 80.4 % of patients were women, 75 % had a technical or university educational level. The main characteristics of the study population are given in Table 1.

**Table 1:** Demographic characteristics of the population

	Medium		Sd
Age	38.7		11.9
Gender (female/male)	201/39		
Married/single	125/115		
Educational level	Primary	Secondary	Technical degree
	14	46	68
			University degree
			112

### Headache characteristics

Migraine without aura was the most common primary headache (77.8 %) prior to MOH. In decreasing order, we found migraine with aura (18.8 %), chronic tension-type headache (15.9 %), and episodic tension-type headache (8.4 %). A minority of patients had chronic migraine as their primary headache. In a minority of patients two types of primary headaches coexisted. Some patients did have more than one headache type. The average age of onset of the primary headache was 17.7 years. Noteworthy, in 52.3 % of subjects, headache started below the age of 16. Headache chronification was related to self-reported stress in 42.7 % of patients.

### Related conditions and habits

Mood and anxiety disorder were common in our patients past medical history: depression was reported in 26.3 %, anxiety in 20.8 %, and insomnia in 43.8 %. Hypertension was present in 12.9 %. Family history was positive for depression in 32.5 %, for alcoholism in 17.5 % and for any type of headache in 73.3 %. Habits findings were notable as regards the use of phosphodiesterase inhibitors beverages on a daily basis: coffee 51.7 %, tea 51.7 %, cola beverages 33.3 % and mate 27.1 %, in most cases in association. 44.2 % of patients drank a moderate amount of alcohol (less than 0.5 L of wine per day) and 26.3 % smoked cigarettes.

### Medications overused

Average length of overuse was 3.9 years (range 1–30 years). In this population, the majority of subjects overused ergotamines (70 %, alone or combined with NSAIDs or caffeine), 33.9 % overused exclusively NSAIDs, 6.3 % combination drugs, and only 5.4 % triptans alone (more than one type of triptans in some cases). The clinical characteristics of patients are given in Table 2.

**Table 2:** Clinical characteristics of the population

	Medium	Sd
Age at onset	17.7	8.5
(years)		
Days of drug intakes/month	21.5	6.9
Headaches days/month	22.4	6.1
Duration of overuse (years)	3.9	4.4

### Use of medical resources

In the year prior to the evaluation, 30.1 % of our patients sought help at the emergency room at least once due to headache, with a range of 1 to 20 visits. 26.8 % consulted a general physician and 37.7 % went for a neurological evaluation at least once. 27.5 % of participants had a head computerized tomography in the last year.

## Discussion

The Latin American section of the COMOESTAS Project represents the largest group of MOH patients published from this region. The demographic profile that has emerged is similar to the published data from developed countries [22], though it is worth noting that both participating medical centers treat patients with private health insurance, which explains the high educational level of the group studied. Our results might therefore not be representative of the general population in our countries. It is necessary to note that Argentina and Chile are the countries with the highest level of education and income in the region; therefore caution must be used when attempting to extrapolate these results to other Latin America countries with certainly.

In agreement with other reports on MOH in other parts of the world, also in this series migraine is by far the most frequently reported primary headache [15, 16, 23]. The average medication overuse time in this series is significantly shorter than previously described [3–5], even though the time passed since the onset of the primary headache is similar. This finding may be related to the exclusion criteria of the COMOESTAS Project, which required naïf MOH subjects (patients without a history of previous failure in detoxification therapy).

In the present series, ergotamine is the drug most frequently overused. This is likely due to the fact that medications containing ergotamine are both the cheapest and most readily available headache treatments in Chile and Argentina, where they are sold without prescription.

Similar to the Katsarava’s studies [8, 9] a relevant percentage of patients in this series made a relation between chronification of pain and stressful situations. At variance, the percentage of subjects with diagnosis of depression or anxiety in our population seems lower than reported in other countries [24]. Even though this could be related to the different methods used for diagnosing depression in the different studies (here we adopted Hospital Anxiety and Depression Scale, HADS), the lower frequency of depression could be linked to the shorter duration of overuse in our series. This raises the possibility that depression and anxiety in MOH may be a consequence of chronic pain, rather than a risk factor.

The tendency to consult a neurologist instead of a primary physician for headaches is characteristic of our study population. Most of our patients are privately insured and tend to choose for themselves who will provide their care. This is a feature of our mixed health insurance system with some patients being served by a public system, while other have access to a private one.

## Conclusion

In conclusion, though little described, MOH is present also in LA, where it affects mostly women, in the most active decades of life. Some differences emerge as regards the demographic and clinical characteristics of MOH in this LA population when compared to Europe or Northern America. What seems more worrying about MOH in Argentina and Chile is that most patients overuse ergotamine, a drug that has been banned from many European countries because of the high risk of toxicity associated to its chronic use. These findings once more underscore the importance of properly diagnose and treat MOH.

## Abbreviations

COMOESTAS: Continuous Monitoring of Medication Overuse Headache in Europe and Latin America
HADS: Hospital Anxiety and Depression Scale
IHS: International Headache Society
MOH: Medication Overuse Headache
